# Intraoperative Bioactivation of Bone Substitutes Using a Surgical Suction Handle: A Prospective Clinical Pilot Study

**DOI:** 10.3390/jfb17050245

**Published:** 2026-05-13

**Authors:** Eleftherios Papaeleftheriou, Andrea Sowislok, Emely Rehage, Alexander Wegner, Marcel Haversath, Melissa Jansen, Marcus Jäger

**Affiliations:** 1 Chair of Orthopedics and Trauma Surgery, University of Duisburg-Essen, 45147 Essen, Germany; e.papaeleftheriou@contilia.de (E.P.); andrea.sowislok@uni-due.de (A.S.);; 2 Department of Orthopedics, Trauma and Reconstructive Surgery, St. Marien Hospital Mülheim a.d. Ruhr, 45468 Mülheim a.d. Ruhr, Germany; 3 Department of Trauma, Orthopedics and Hand Surgery, Klinikum Wolfsburg, 38440 Wolfsburg, Germany; alexander.wegner@klinikum-wolfsburg.de; 4 Centre Arthroscopy and Arthroplasty, Städtisches Krankenhaus Nettetal GmbH, 41334 Nettetal, Germany; m.haversath@krankenhaus-nettetal.de; 5 Institute of Cognitive Science, University of Osnabrück, 49090 Osnabrück, Germany; mejansen@uni-osnabrueck.de; 6 Department of Orthopaedics, Trauma and Reconstructive Surgery, Katholisches Klinikum Essen Philippusstift, 45355 Essen, Germany

**Keywords:** bioactivation, bone healing, bone substitute, regeneration, scaffold, suction handle, vacuum

## Abstract

Critical size bone defects (CSBD) remain a major challenge in orthopedic surgery. Autologous bone grafting is considered the gold standard but is limited by restricted availability and significant donor-site morbidity. Synthetic bone substitutes offer an alternative; however, these materials are avital and lack osteoinductive properties. This study evaluated whether intraoperative bioactivation of bone substitutes using a surgical suction handle can safely enhance their regenerative potential. Fifty patients with CSBD, non-unions, or high-risk defects were enrolled, and calcium phosphate-based ceramics were intraoperatively coated with autologous tissue via a surgical suction handle and implanted into the defects. Clinical outcomes—including pain, range of motion, and wound healing—were scored using a standardized system, with all patients achieving results in the “excellent” range (10–13 points). Radiographic follow-up showed progressive cortical and extracortical bone formation in all patients. Surgeons reported high ease-of-use for the device, and no device-related complications occurred. Although biomaterial resorption was incomplete in some cases (36% with <75% resorption at six months), no patient required revision surgery. Our data indicate that intraoperative bioactivation of bone substitutes using a surgical suction handle is safe, feasible, and promotes local bone regeneration, providing a minimally invasive and practical approach to enhance the performance of synthetic grafts in challenging defects.

## 1. Introduction

Critical size osseous defects due to trauma, total joint revisions, after infection, or tumor resections remain an unsolved challenge for the orthopedic surgeon and are a high social and economic burden for the patient [1,2]. Moreover, joint preservation in advanced avascular osteonecrosis as a special type of bone defect is challenging and frequently requires joint replacement even among the younger population [3].

Bone defects as well as delayed bone healing are not only categorized by different causes, risk factors, or their anatomic region, but also by a high variability in extent and geometry. This complicates the assessment of comparable clinical data and thus evidence-based recommendations. Therefore, even when following orthopedic standards and guidelines, individual parameters and thus treatment are individualized in most of these patients [4].

Due to its high regenerative potential, the low risk of infection, or adverse immuno-reactions, autologous bone grafting including its marrow is still the gold standard for the surgical treatment of large bone defects, pseudarthrosis, or delayed bone healing. However, autologous bone grafting is associated with relevant risks and side effects [5]. Substantially, these include donor-site morbidity and prolonged duration of surgery. Additional disadvantages of autologous bone grafting include the poor quality of the graft, especially among the elderly population. Moreover, the amount of autologous tissue is limited, making it sometimes impossible to cover the whole area on a large-scale bone defect.

Useful alternatives to autologous bone grafting include the application of allo-/xenografts (e.g., XABC) or the use of synthetic bone substitute materials [6,7]. Most of the latter consist of highly porous calcium phosphate (CaP) ceramics or material combinations (e.g., hydroxyapatite (HA), tricalcium phosphate, calcium carbonate, calcium sulfate, calcium sodium phosphosilicate). The high porosity and large surface area of these materials allow small diffusion distances for cellular nutrients and thus promote local tissue ingrowth [8].

However, if applied in solid states (granules, stripes, blocks etc.) porous calcium-phosphates require complete wetting prior to application as described in the instructions for use (IFU) of most legal manufacturers or distributors. Otherwise, local pH changes can occur leading to a lack in biocompatibility of the material [9]. Another characteristic of bioceramics is that all these materials are avital, serving only as a scaffold to guide local cells and thus lacking intrinsic osteopromotive properties (osteoinduction).

To improve the osteopromotive properties of bone substitute materials, several pathways have been studied over the years including the application of autologous bone marrow or its concentrate [10,11,12,13], combination with growth factors (e.g., bone morphogenetic protein (BMP)-2) [14,15,16,17], or autologous cytokine cocktails, including platelet-rich plasma (PRP), fibrin (PRF), or extracellular vesicles (EVs) [18,19,20,21,22,23]. Moreover, some data from the literature suggest that surgical site-released tissue has a strong osteopromotive potential since it contains significant amounts of growth factors and cytokines [24,25,26]. In a pilot study, we applied bone substitute materials to an internal filter of a surgical suction handle, and found that the negative pressure within the handle impregnates the inserted bone substitute sufficiently with surgical site-released tissue and fluid (blood, cytokines, small tissue fragments) during orthopedic surgery [24,27]. Moreover, we demonstrated strong protein binding onto calcium phosphate-based bone substitutes, acting as a biological glue and interface for cellular binding [28].

Building on these preclinical observations, the aim of this study was to investigate the clinical applicability, safety, and regenerative potential of the surgical suction handle as a novel tool for intraoperative bioactivation of bone substitute materials.

## 2. Materials and Methods

Patients: In a prospective, non-randomized four-center study, 50 patients with insufficient bone healing were documented over a minimum follow-up period of 6 months. Detailed information on patient characteristics, diagnoses, applied scaffold materials, surgical procedures, and healing outcomes is provided in Supporting Table S1. Patient inclusion criteria were critical size bone defects with an indication of auto- or allografting, pseudarthrosis, and patients at high risk of delayed or non-healing of the bone > 50% based on relevant scientific literature and international guidelines. The exclusion criteria comprised vertebral body defects (according to IFU of the system), chronic or acute systemic infections, consuming diseases (active neoplasm, autoimmune disorders, acquired immunodeficiency syndrome), or unwilling to take part in the study. The study protocol followed international standards such as the Declaration of Helsinki in its present version. Prior to participation in the study, written informed consent was obtained from all patients. Due to the poor prognostic outcome of the underlying bone defect, the patients were informed of the limited predictability of the outcome and the nature of healing attempt before treatment. The study was approved by an Institutional Review Board/Ethics Committee (#Bo/35/2025-Studie-01/clinical registration code “NCT07562165”).

Materials: Bone defects were treated by bone substitute material (synthetic or allograft) activated and coated by autologous tissue under vacuum in a suction handle with an integrated filter (BoneFlo^®^, TissueFlow GmbH, Essen, Germany).

**Figure 1:**
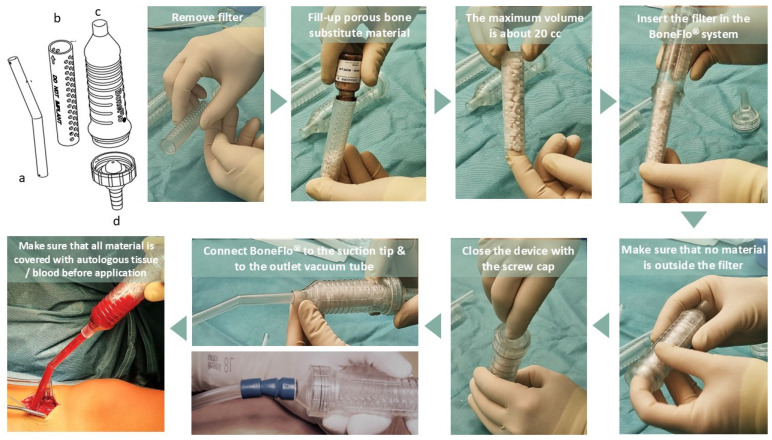
Application steps using an PMMA-based surgical suction handle (BoneFlo^®^) as a tissue collector to coat bone substitutes by autologous tissue released at the operation site (off-label). The device consists of a suction tip (a), an internal filter with multiple pores (b), a plastic case including an inlet opening (c) and a screw cap with an outlet opening (d). The bone substitute material is inserted into the internal filter of the suction handle prior to surgery. During surgery, the device was applied only after the surgeon had confirmed complete resection of all pathological tissue (e.g., pseudarthrosis, debris). The minimal, obligatory suction time for biomaterial covering was 10 min. Moreover, all surgeons were instructed to ensure that all parts of the bone substitute material were fully impregnated by autologous tissue prior to application (visually).

All participating surgeons were instructed in the use of the device using a manual as well as practical hands-on training before application (Figure 1). The vacuum applied to the suction set device was within the typical range in orthopedic surgery between 250–350 mmHg. A standardized survey served to document side effects, the performance of the system, and user satisfaction:

The following bone substitute materials were used in this study.

- Highly porous beta-tricalcium phosphate (TCP) granules, 1000–2000, 3000–5000 and 5000–8000 µm (Cerasorb^®^ M, CURASAN, Kleinostheim, Germany)
- Porcine collagen I foam including embedded TCP granules (85 wt.%) (Cerasorb^®^ Flexible Foam Strip, CURASAN, Kleinostheim, Germany)
- Injectable synthetic bone void filler (40% HA, 60% CaSO_4_, gentamicin sulfate (Cerament^®^ G, BoneSupport, Lund, Sweden)
- Cortico-spongious femoral head allografts (German Institute for Cell and Tissue Replacement, DIZG, Berlin, Germany)
- Triosite^®^ Bioactive Ceramic Bone Graft Substitute (60% HA, 40% TCP, Zimmer Biomet, Warsaw, IN, USA)
- b.Bone Granules 2–7.1 mm (HA: 80 ± 10%, beta-TCP: 15 ± 10% TCP, Ca/P: 1.6 ± 0.1, total porosity ≥45%, interconnection ≥45%, macropores (>10 µm): >30%, micropores (<10 µm): >30%, GreenBone Ortho S.p.A., Faenza, Italy.

Surgical procedure: Surgical procedure included debridement of the bone defect area, curettage, and, if required, additional stabilization by osteosynthesis or endoprosthesis. Afterwards, the coated and thus bioactivated bone substitute material was applied to the local bone defect using standard surgical instruments (forceps, spoon, plunger). To prevent dislocation of the bone substitute material from the implantation site to surrounding soft tissue, a conventional sterile, water-insoluble porcine gelatin absorbable sponge was used as a barrier (Spongostan™ Standard, Fa. Ethicon, Johnson & Johnson Medical GmbH, Norderstedt, Germany) in cortical defects. Moreover, there was no pre-treatment of the implantation situs by any disinfectants.

Intraoperative device evaluation: After surgery, all surgeons completed a questionnaire assessing handling and usability of the device. The evaluation was performed using a school grading system (1 = very good to 6 = poor) across several parameters. Mean scores for all parameters were calculated to quantify ease of use and overall performance of the device.

Clinical outcome scoring: A clinical outcome scoring system was developed to quantify postoperative patient outcomes. The system assessed three parameters: pain, range of motion of adjacent joints proximal and distal to the lesion (active and passive), and wound healing. Each parameter was assigned a score, with higher values indicating better outcomes. Total scores were classified as Excellent (10–13), Moderate (6–9), or Poor (1–5) (Table 1).

Clinical and radiological outcome scores were assessed at predefined intervals (2–3 weeks, 6 weeks, 12 weeks, 6 months). In addition, all complications, including revision surgeries, infections, wound healing disorders, and other adverse events, were documented.

Bone formation was assessed using a modified mRUST score [29,30]. Since no established classification systems exist for extracortical bone formation (ECB), ECB was recorded in analogy to mRUST scoring (Table 1), while biomaterial resorption was documented using a modified Bergland score [31] (Table 2). This adapted scoring provides a structured, semi-quantitative assessment, but results should be interpreted with caution due to differences between cortical and extracortical bone formation and potential variations in defect size and biomaterial type. 

## 3. Results

### 3.1. Patient Demographics and Bone Substitute Application

**Figure 2:**
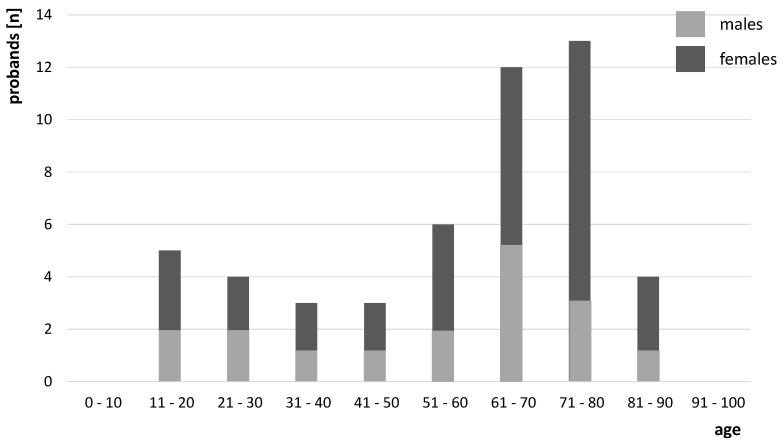
Age and sex distribution of the study cohort.

The study included 50 patients (33 females, 17 males) aged 11–90 years (mean 57.5 years, median 65 years, SD 21.6) with a minimum follow-up of six months (Figure 2).

Patients were treated across four centers, with the majority recruited at the primary center (Center I, 45 patients), which served as the main study site with the largest patient population and most active recruiting surgeons. Smaller numbers of patients were recruited at Centers II–IV due to their lower caseloads during the study period. Seven surgeons participated, treating between one and 29 patients each (Table 3). The most applied bone substitute material was porous beta-TCP granules (*n* = 34, 68.0%), followed by TCP-collagen scaffolds (*n* = 11, 22.0%), allografts (*n* = 6, 12.0%), and HA/TCP composites (Triosite™, *n* = 3, 6%). Six patients (12.0%) received combinations of different bone substitutes, and in one case bone morphogenic protein 2 (12 mg, InductOs™, Medtronic GmbH, Meerbusch, Germany) was additionally applied (Table 3).

**Figure 3:**
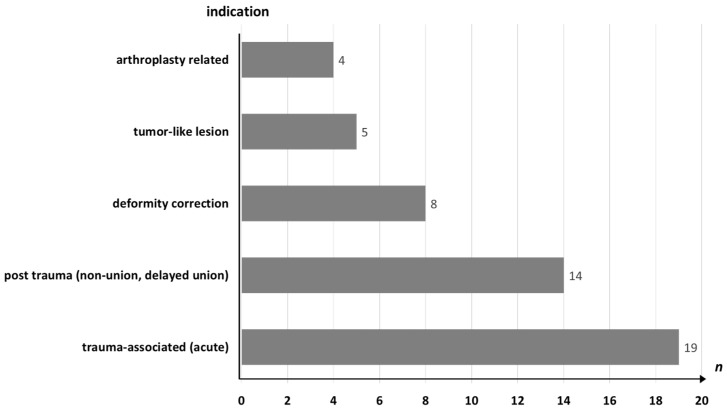
Distribution of surgical indications in the study cohort.

Most patients underwent surgery due to acute trauma (38%), while 14 (28%) patients were treated for complications following prior trauma, such as non-union or pseudarthrosis. Other indications included arthroplasty-related issues, and tumor-like lesions (Figure 3).

### 3.2. Intraoperative Handling and Safety of the Device

**Figure 4:**
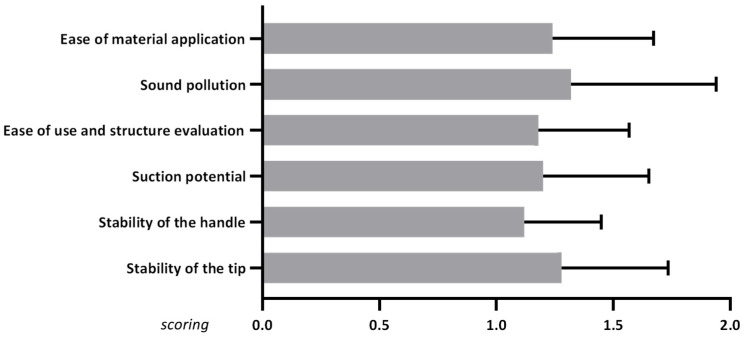
Surgeon ratings of the intraoperative performance of BoneFlo^®^ across multiple parameters.

All seven surgeons rated the surgical suction handle under this off-label scenario (bioactivation of bone substitutes) as “easy to handle”. Also, noise pollution was low, and suction performance was not significantly affected by the biomaterial. Furthermore, no device-related complications, malfunctions, or other negative side effects were observed. In two cases, where TCP granules smaller than 3000 µm were applied, minor material loss occurred due to the large pore size of the inner filter. The procedure did not increase operative time, as the bioactivation of the scaffold by vacuum was performed in parallel with conventional suction, replacing the typical time required for wetting the bone substitutes. Moreover, the device received consistently high intraoperative ratings across parameters including connection stability to the suction tip and tubing, handling, and grip (Figure 4).

### 3.3. Postoperative Clinical Outcomes and Healing

The clinical outcome scoring system assessed pain, wound healing, and range of motion using predefined scores. Of 50 patients that took part in the study, none of them required a revision surgery, and all patients achieved excellent healing outcomes. None of them showed moderate or poor healing.

Postoperative examinations demonstrated uneventful wound healing in all patients, with no prolonged wound secretion, delayed soft tissue healing, or infections. Early mobilization and weight-bearing according to the surgeon’s recommendations were successfully achieved by all patients.

**Figure 5:**
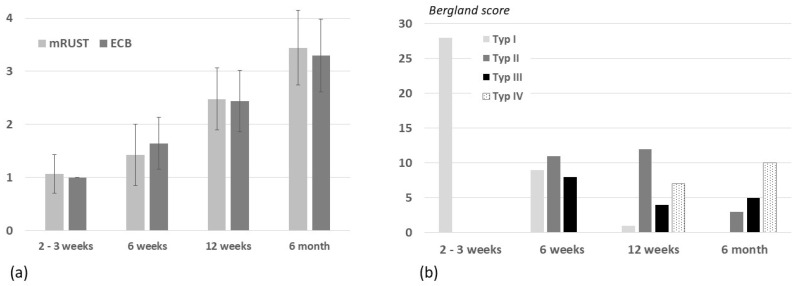
(**a**) Cortical (mRUST) and extracortical (ECB) bone formation in patients during 6-month follow-up (no bone formation: 1, increased density: 2, subtotal bridging: 3, complete bridging/healing: 4). (**b**) Biomaterial resorption over a 6-month period according to modified Bergland classification (type I: 0 to 25%, type II: 25 to 50%, type III: 50 to 75%, type IV: 75–100% of resorption).

New bone formation and bone healing documented on radiographs in two planes showed progressive mineralization. At the latest follow-up (6 months), all treated patients showed healing of the bone defect (Figure 5a). Although biomaterial resorption proceeds over the 6 months, we found relevant residues of biomaterials in radiographs due to a lack of resorption and degradation of these materials (Figure 5b).

### 3.4. Representative Clinical Cases Illustrate Outcomes Across Different Age Groups and Defect Types

**Figure 6:**
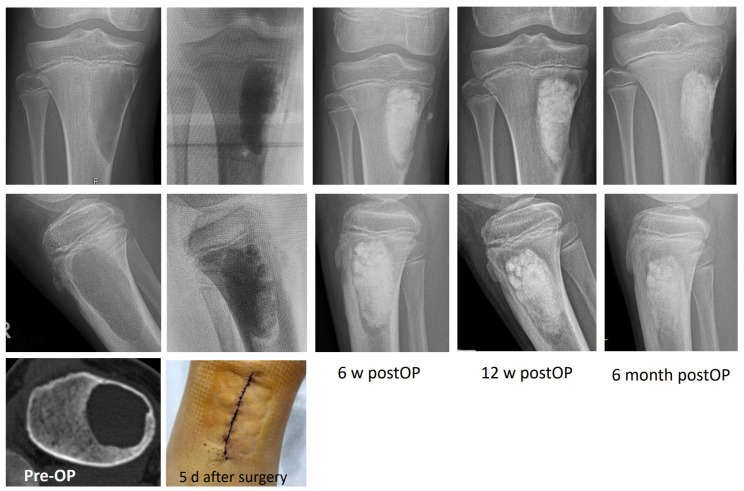
Radiographic 6-month follow-up of a 11-year-old male patient with clinically apparent solitary bone cyst. Due to the fracture risk by eggshell-thin lateral cortical bone and the performance of contact sports, the cyst was curetted by a cortical window and treated by activated TCP granules. Follow-up showed an uneventful wound and complete bone healing, including remodeling of the cortical bone and partial resorption of the biomaterial. The latest clinical follow-up 1 year after surgery showed a fully active and pain-free patient.

**Figure 7:**
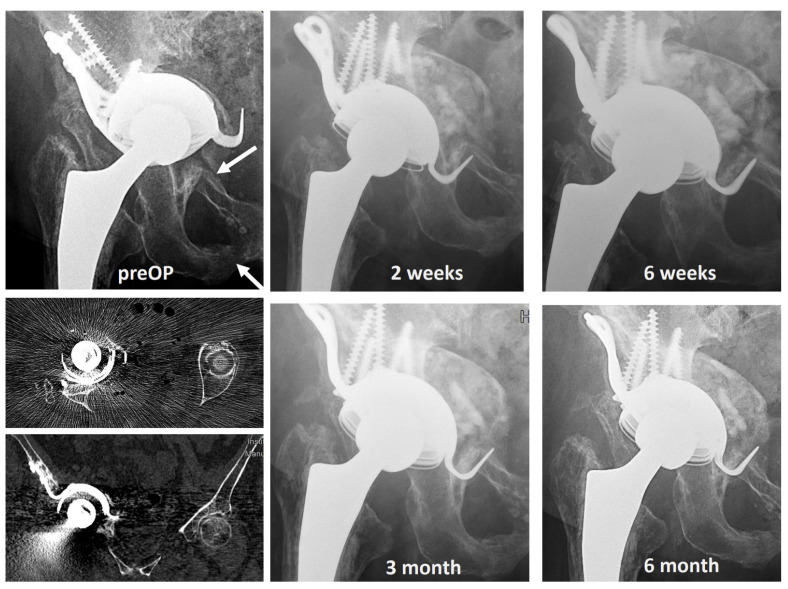
Radiographic 6-month follow-up of a 76-year-old female with a critical size pelvic defect (pelvic discontinuity) after failed revision cup and fracture of the os ischium and superior pubic ramus. The arrows show the fracture gap. The large pelvic defect was treated by bioactivated 2 × 20 cl TCP granules (5000–8000 µm) as well as an allograft, and the hip joint was reconstructed by a cementless, modular revision titanium cup (MRP, Fa. Brehm, Germany). Three months postoperative the patient achieved full weight-bearing. The latest follow-up showed a healed fracture and an osseous integrated cup.

**Figure 8:**
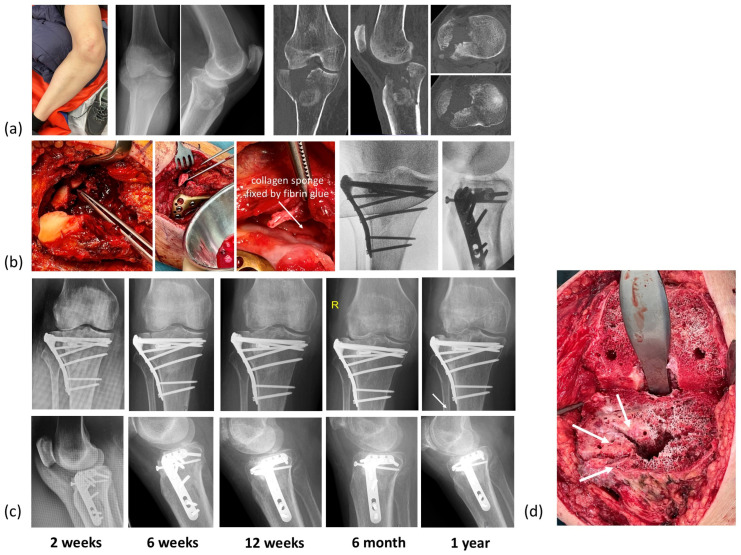
Comminuted tibia plateau fracture with valgus deformity, significant bone loss, and frontal instability in a 64-year-old female. (**a**) Clinical picture and radiographs/CT scan. (**b**) The intraoperative procedure included restoration of the joint line using an allograft block (50% of a femoral head) and bioactivated TCP granules. The cartilage defect zone was covered by a fibrin glue fixed collagen sponge to obtain a barrier between bone substitute materials and the intraarticular joint space. The fracture was stabilized by a tibia plateau plate and locking screws (Fa. Synthes, USA). (**c**) The postoperative follow-up over 1 year showed progressive healing of the fracture with a solid bone stock in case of later joint replacement. A small defined subchondral defect zone remains (arrow). (**d**) Due to post-traumatic osteoarthritis the patient underwent total knee replacement 1.5 years after ORIF. The intraoperative situs showed solid bone formation within the former defect zone (arrows).

An 11-year-old patient with a solitary bone cyst achieved complete healing (Figure 6), and a 76-year-old patient with a large pelvic defect showed fracture healing and osseointegration of the revision cup (Figure 7). Also, a severe bone defect due to tibial plateau fracture healed well within the follow-up of 6 months (Figure 8).

## 4. Discussion

**Figure 9:**
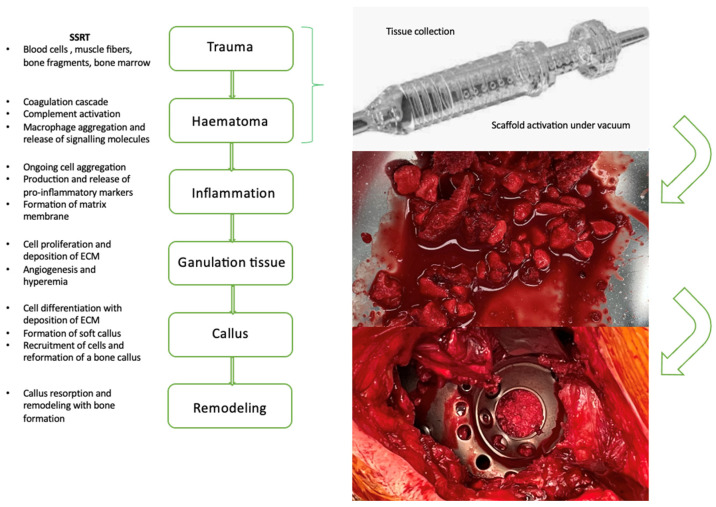
The inflammatory and regenerative process leading to bone healing. The BoneFlo^®^ surgical suction device is shown on the far right, indicating its application during the early phase (trauma and hematoma) to collect surgical site-released tissue (SSRT) and bioactivate the scaffold in situ. The device captures tissue fragments, blood, and cytokines at the surgical site, which then coat the scaffold to enhance osteogenic potential during the subsequent healing phases.

Bone healing and remodeling is a complex cascade of cellular and molecular events (Figure 9) [32]. Trauma and/or surgical intervention triggers tissue damage and bleeding, activating coagulation and complement pathways to form a fibrin-rich hematoma. Local macrophages orchestrate the early response by clearing necrotic tissue and secreting cytokines and growth factors, including BMP2 and others, which promote bone formation [33,34]. The subsequent inflammatory phase recruits additional progenitor cells via chemotaxis and transendothelial migration. Mesenchymal progenitor cells (MSCs) deposit an extracellular matrix and secrete cytokines that promote angiogenesis. MSC differentiation into pre-osteoblasts supports formation of a provisional callus, which is gradually remodeled into mature bone [35]. Here growth factors such as VEGF [36,37,38] and PDGF [39] play a key role throughout this process.

Autologous tissue-based augmentation strategies to enhance bone regeneration are well established in orthopedic surgery, as exemplified by vacuum techniques such as the reamer–irrigator–aspirator (RIA) or other lower invasive harvesting systems (e.g., Avitus™, Zimmer Biomet, Warsaw, IN, USA) [40]. However, although these techniques are aiming to prevent donor-site morbidity due to bone grafting, they still are more or less invasive [41,42]. Building on non-invasive and time-saving principles, we investigated a conceptually related but technically simplified approach: the intraoperative, single-step biologization of synthetic bone substitutes using surgical site-released tissue (SSRT) collected via a surgical suction handle during orthopedic surgery. In our case series, we applied predominantly highly porous β-TCP granules, which are osteoconductive but not osteoinductive. Remarkably, even in critical-sized defects and compromised healing conditions, encouraging outcomes were observed in our cohort. Although causality cannot be definitively established and the study lacked a control group, these results support the hypothesis that intraoperative exposure to SSRT may enhance the regenerative potential of otherwise inert scaffolds.

For safety reasons, and in contrast to Wu et al. [40], we did not apply the vacuum system in infection or tumor cases. However, the BoneFlo system was successfully used in enchondroma. Here, peripheral venous blood served to cover TCP granules to prevent re-transplantation of enchondroma tissue fragments (unpublished data). Since SSRT reflects the immediate microenvironment of early bone healing and contains a complex mixture of tissue fragments, MSCs, cytokines, and growth factors [25,26,28,43,44], we hypothesize that SSRT is a crucial initiator for bone regeneration (Figure 9). Immediately after implantation, scaffold surfaces rapidly adsorb this autologous mixture, forming a bioactive layer that promotes cell adhesion, modulates inflammatory signaling, stimulates angiogenesis, and initiates osteogenic cascades, all processes that are critical for successful osseointegration [45,46]. SSRT also contains higher concentrations of pro-angiogenic and osteogenic mediators such as VEGF and PDGF compared with peripheral venous blood [24], and in vitro studies revealed upregulation of osteogenic marker genes and a distinct microRNA profile associated with neovascularization, matrix mineralization, and osteoblast differentiation [26].

Enrichment of bone substitutes with SSRT via a vacuum filter requires no additional surgical steps or instruments, allowing seamless integration into surgical routine. This was confirmed intraoperatively: all surgeons reported high satisfaction, describing the suction handle as intuitive and safe to use. No device-related complications occurred, and because bioactivation was performed in parallel with standard suction, operative time was unaffected. Minor material loss with very small TCP granules was observed, suggesting potential for future technical refinement.

Overall, these findings demonstrate that intraoperative SSRT enrichment is both technically feasible and readily integrated into clinical practice. While these results are encouraging, several limitations must be considered: the study was non-randomized, lacked a control group, and patients were heterogeneous in defect type, location, and individual risk factors. Therefore, it is not possible to definitively attribute the observed positive outcomes solely to SSRT coating, and efficacy claims should be interpreted with caution. Nevertheless, the consistency of clinical and radiographic outcomes across centers, combined with mechanistic rationale and supportive experimental evidence, suggests that intraoperative SSRT coating is a feasible and safe adjunct to enhance the regenerative potential of bone substitutes.

## 5. Conclusions

In this clinical pilot study, the intraoperative application of SSRT-coated bone substitutes using the BoneFlo^®^ surgical suction handle was safe, feasible, and well tolerated across all study sites. Clinical outcomes and radiographic assessments demonstrated progressive bone formation and uneventful wound healing in patients with challenging bone defects. While the specific mechanistic contribution of individual SSRT components remains to be elucidated, our data support the concept that intraoperative bioactivation enhances the regenerative potential of synthetic bone substitutes. These findings suggest that the BoneFlo^®^ system can serve as a practical adjunct in orthopedic surgery to improve bone healing, complementing established treatment strategies.

## Figures and Tables

**Table 1:** Clinical outcome scoring system Key: 10–13 Excellent, 6–9 Moderate, 1–5 Poor.

Pain		Range of Motion				Wound Healing	
		Active		Passive			
4	No pain	3	Full 100%	3	Full 100%	3	Complete
3	Mild pain	2	Moderate <50%	2	Moderate <50%	2	Delayed
2	Moderate pain	1	Minimal 0–50%	1	Minimal 0–50%	1	Dehiscence
1	Severe pain	0	None 0%	0	None 0%	0	No healing

**Table 2:** Radiological scoring systems used to assess bone healing and biomaterial resorption during follow-up. Cortical bone formation was evaluated using a modified mRUST score [29,30]. Due to missing classification systems, we transferred the mRUST to extracortical bone regeneration (ECB) including intra- and extramedullary bone formation. Biomaterial resorption (allograft or synthetic bone substitutes) was assessed using a modified Bergland score [31] on two-plane radiographs and CT scans if available.

Cortical Bone Formation (mRust Score)	Extracortical Bone Formation (ECB Score)	Biomaterial Resorption (Bergland Score)
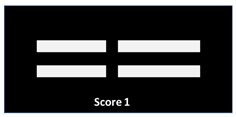	no formation: 1	Type I: 0 to 25%
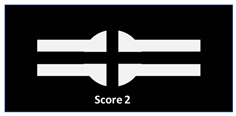	increased density: 2	Type II: 25 to 50% of
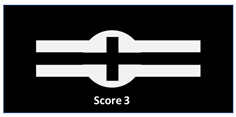	subtotal bridging: 3	Type III: 50 to 75% of resorption
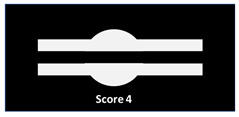	complete healing: 4	Type IV: 75 to 100%

**Table 3:** Distribution of patients across participating centers, number of involved surgeons (# 1−7), and types of bone substitute materials applied. “-” indicates not available.

Centers		I	II	III	IV
**Number of patients**		45	1	2	2
**Number of surgeons**		4	1	1	1
Patients per surgeon: #1 (29), #2 (6), #3 (7), #4 (2), #5 (1), #6 (2), #7 (1)					
**Applied material**	TCP granules	32	1	1	-
	TCP collagen scaffold	10	-	1	-
	HA/TCP	-	1	-	2
	HA/CaSO_4_ cement + gentamycin or vancomycin	1	-	1	-
	Allograft (spongious)	6	-	-	-
	BMP2	-	-	1	-

## Data Availability

The original contributions presented in this study are included in the article/Supplementary Material. Further inquiries can be directed to the corresponding author.

## Supplementary Materials

The following supporting information can be downloaded at: https://www.mdpi.com/article/10.3390/jfb17050245/s1, Table S1: Clinical characteristics and surgical procedures of the included patients. Key for diagnosis category: 1: arthroplasty, 2: tumor like lesions, 3: deformity correction, 4: post-trauma, 5: trauma associated.

## References

[1] O’Hara N.N., Isaac M., Slobogean G.P., Klazinga N.S. (2020). The socioeconomic impact of orthopaedic trauma: A systematic review and meta-analysis. PLoS ONE.

[2] Collaborators G.B.D.F. (2021). Global, regional, and national burden of bone fractures in 204 countries and territories, 1990–2019: A systematic analysis from the global burden of disease study 2019. Lancet Healthy Longev..

[3] George G., Lane J.M. (2022). Osteonecrosis of the femoral head. J. Am. Acad. Orthop. Surg. Glob. Res. Rev..

[4] Li L., Patel M., Zhang L. (2026). A review of bone fracture healing modelling: From mechanobiological theory to personalized rehabilitation protocols. Bone.

[5] Schmidt A.H. (2021). Autologous bone graft: Is it still the gold standard?. Injury.

[6] Feroz S., Cathro P., Ivanovski S., Muhammad N. (2023). Biomimetic bone grafts and substitutes: A review of recent advancements and applications. Biomed. Eng. Adv..

[7] Bondi M., Piotto L., Pizzoli A. (2025). State of the art for bone substitutes. J. Orthop. Orthop. Surg..

[8] Bohner M., Le Santoni B.G., Döbelin N. (2020). β-tricalcium phosphate for bone substitution: Synthesis and properties. Acta Biomater..

[9] Tilkeridis K., Touzopoulos P., Ververidis A., Christodoulou S., Kazakos S., Drosos G. (2014). Use of demineralized bone matrix in spinal fusion. World J. Orthop..

[10] Van Vugt T.A.G., Geurts J.A.P., Blokhuis T.J. (2021). Treatment of infected tibial non-unions using a bmac and s53p4 bag combination for reconstruction of segmental bone defects: A clinical case series. Injury.

[11] Stanovici J., Le Nail L.R., Brennan M.A., Vidal L., Trichet V., Rosset P., Layrolle P. (2016). Bone regeneration strategies with bone marrow stromal cells in orthopaedic surgery. Curr. Res. Transl. Med..

[12] Vulcano E., Murena L., Cherubino P., Falvo D.A., Rossi A., Baj A., Toniolo A. (2012). Treatment of severe post-traumatic bone defects with autologous stem cells loaded on allogeneic scaffolds. Surg. Technol. Int..

[13] Mavrogenis A.F., Karampikas V., Zikopoulos A., Sioutis S., Mastrokalos D., Koulalis D., Scarlat M.M., Hernigou P. (2023). Orthobiologics: A review. Int. Orthop..

[14] Wang G., Alagboso F.I., Walter N., Baertl S., Brochhausen C., Docheva D., Rupp M., Alt V. (2022). Bone regeneration after marginal bone resection in two-stage treatment of chronic long bone infection-a combined histopathological and clinical pilot study. Injury.

[15] Rosenberg M., Shilo D., Galperin L., Capucha T., Tarabieh K., Rachmiel A., Segal E. (2019). Bone morphogenic protein 2-loaded porous silicon carriers for osteoinductive implants. Pharmaceutics.

[16] Betz O.B., Betz V.M., Schroder C., Penzkofer R., Gottlinger M., Mayer-Wagner S., Augat P., Jansson V., Muller P.E. (2013). Repair of large segmental bone defects: Bmp-2 gene activated muscle grafts vs. Autologous bone grafting. BMC Biotechnol..

[17] Starman J.S., Bosse M.J., Cates C.A., Norton H.J. (2012). Recombinant human bone morphogenetic protein-2 use in the off-label treatment of nonunions and acute fractures: A retrospective review. J. Trauma Acute Care Surg..

[18] Khazaei F., Rezakhani L., Alizadeh M., Mahdavian E., Khazaei M. (2023). Exosomes and exosome-loaded scaffolds: Characterization and application in modern regenerative medicine. Tissue Cell.

[19] Gholami Farashah M.S., Javadi M., Mohammadi A., Soleimani Rad J., Shakouri S.K., Roshangar L. (2022). Bone marrow mesenchymal stem cell’s exosomes as key nanoparticles in osteogenesis and bone regeneration: Specific capacity based on cell type. Mol. Biol. Rep..

[20] Leung K.S., Shirazi S., Cooper L.F., Ravindran S. (2022). Biomaterials and extracellular vesicle delivery: Current status, applications and challenges. Cells.

[21] Rana N., Suliman S., Al-Sharabi N., Mustafa K. (2022). Extracellular vesicles derived from primed mesenchymal stromal cells loaded on biphasic calcium phosphate biomaterial exhibit enhanced macrophage polarization. Cells.

[22] Sun Y., Feng Y., Zhang C.Q., Chen S.B., Cheng X.G. (2010). The regenerative effect of platelet-rich plasma on healing in large osteochondral defects. Int. Orthop..

[23] Sarkar M.R., Augat P., Shefelbine S.J., Schorlemmer S., Huber-Lang M., Claes L., Kinzl L., Ignatius A. (2006). Bone formation in a long bone defect model using a platelet-rich plasma-loaded collagen scaffold. Biomaterials.

[24] Busch A., Herten M., Haversath M., Kaiser C., Brandau S., Jager M. (2020). Ceramic scaffolds in a vacuum suction handle for intraoperative stromal cell enrichment. Int. J. Mol. Sci..

[25] Henze K., Herten M., Haversath M., Busch A., Brandau S., Hackel A., Flohe S.B., Jager M. (2019). Surgical vacuum filter-derived stromal cells are superior in proliferation to human bone marrow aspirate. Stem Cell Res. Ther..

[26] Groven R.V.M., Blokhuis J.T., Poeze M., van Griensven M., Blokhuis T.J. (2023). Surgical suction filter-derived bone graft displays osteogenic mirna and mrna patterns. Eur. J. Trauma Emerg. Surg..

[27] Jager M., Busch A., Sowislok A. (2022). Bioactivation of scaffolds in osteonecrosis. Orthopadie.

[28] Sowislok A., Gruber G., Kaschani F., Kaiser M., Papaeleftheriou E., Jager M. (2025). Intraoperative biologization of beta-tcp and pcl-tcp by autologous proteins. J. Funct. Biomater..

[29] Leow J.M., Clement N.D., Simpson A. (2020). Application of the radiographic union scale for tibial fractures (rust): Assessment of healing rate and time of tibial fractures managed with intramedullary nailing. Orthop. Traumatol. Surg. Res..

[30] Leow J.M., Clement N.D., Tawonsawatruk T., Simpson C.J., Simpson A.H. (2016). The radiographic union scale in tibial (rust) fractures: Reliability of the outcome measure at an independent centre. Bone Jt. Res..

[31] Bergland O., Semb G., Abyholm F.E. (1986). Elimination of the residual alveolar cleft by secondary bone grafting and subsequent orthodontic treatment. Cleft Palate J..

[32] Einhorn T.A., Gerstenfeld L.C. (2015). Fracture healing: Mechanisms and interventions. Nat. Rev. Rheumatol..

[33] Wei F., Zhou Y., Wang J., Liu C., Xiao Y. (2018). The immunomodulatory role of bmp-2 on macrophages to accelerate osteogenesis. Tissue Eng. Part A.

[34] Frade B.B., Dias R.B., Gemini Piperni S., Bonfim D.C. (2023). The role of macrophages in fracture healing: A narrative review of the recent updates and therapeutic perspectives. Stem Cell Investig..

[35] Wang Z., Ren L., Li Z., Qiu Q., Wang H., Huang X., Ma D. (2025). Impact of different cell types on the osteogenic differentiation process of mesenchymal stem cells. Stem Cells Int..

[36] Hu K., Olsen B.R. (2017). Vascular endothelial growth factor control mechanisms in skeletal growth and repair. Dev. Dyn..

[37] Hu K., Olsen B.R. (2016). The roles of vascular endothelial growth factor in bone repair and regeneration. Bone.

[38] Hu K., Olsen B.R. (2016). Osteoblast-derived vegf regulates osteoblast differentiation and bone formation during bone repair. J. Clin. Investig..

[39] Novak S., Madunic J., Shum L., Vucetic M., Wang X., Tanigawa H., Ghosh M., Sanjay A., Kalajzic I. (2023). Pdgf inhibits bmp2-induced bone healing. npj Regen. Med..

[40] Wu K.A., Shenoy D., Sachs E., Somarelli J.A., Pean C., DeBaun M., Brigman B.E., Visgauss J.D., Eward W.C. (2024). Exploring versatile applications of a vacuum-assisted bone harvester in orthopedic surgery. BMC Musculoskelet. Disord..

[41] Metsemakers W.J., Claes G., Terryn P.J., Belmans A., Hoekstra H., Nijs S. (2019). Reamer-irrigator-aspirator bone graft harvesting for treatment of segmental bone loss: Analysis of defect volume as independent risk factor for failure. Eur. J. Trauma Emerg. Surg..

[42] Nauth A., Lane J., Watson J.T., Giannoudis P. (2015). Bone graft substitution and augmentation. J. Orthop. Trauma.

[43] Rehage E., Sowislok A., Busch A., Papaeleftheriou E., Jansen M., Jager M. (2023). Surgical site-released tissue is potent to generate bone onto tcp and pcl-tcp scaffolds in vitro. Int. J. Mol. Sci..

[44] Lodewijks A.J.L., Warin M.M.R., van der Broeck L.C.A., Fois M.G., Lopez-Iglesias C., Blokhuis T.J., van Griensven M., Poeze M. (2025). Long-term follow-up of patients with large segmental bone defects treated with 3d-printed polycaprolactone/tricalcium phosphate scaffolds. Eur. J. Trauma Emerg. Surg..

[45] Jager M., Jennissen H.P., Haversath M., Busch A., Grupp T., Sowislok A., Herten M. (2019). Intrasurgical protein layer on titanium arthroplasty explants: From the big twelve to the implant proteome. Proteom. Clin. Appl..

[46] Jager M., Latosinska A., Herten M., Busch A., Grupp T., Sowislok A. (2022). The implant proteome-the right surgical glue to fix titanium implants in situ. J. Funct. Biomater..

