# Supplementary material for: Intraoperative Bioactivation of Bone Substitutes Using a Surgical Suction Handle: A Prospective Clinical Pilot Study
Source: J Funct Biomater. 2026 May 13;17(5):245. doi: 10.3390/jfb17050245 (PMC13208171; doi:10.3390/jfb17050245)
Supplement: Supplementary file 1 [file jfb-17-00245-s001.zip › jfb-4211011-supplementary.pdf]

Supplementary Material

Surgical suction handle - an innovative tool to activate bone substitutes

E. Papaeleftheriou *et. al.*

Supporting Table S1: Clinical characteristics and surgical procedures of the included patients. Key for diagnosis category: 1: arthroplasty, 2: tumor like lesions, 3: deformity correction, 4: post-trauma, 5: trauma associated.

Nr. In relation to diagnosis	Proband ID	Diagnosis	Scaffold	Procedure	Healing Completed (+/-)	Revisions (+/-)
2	1	Solitary subchondral cyst (tibia)	TCP granules (3 x 10 cc Cerasorb® M 5000-8000µm)	Curettage of the cystic lesion and filling with TCP	+	
5	2	Fx of the tibial plateau	TCP granules (1 x 10 cc Cerasorb® M 3000-5000µm)	ORIF (plate/screws)	+	
3	3	Genu varu and chondromalacia patellae	TCP granules (1 x 10 cc Cerasorb® M 3000-5000µm) augmented by allograft bone chips	Valgus osteotomy	+	
1	4	Acetabular defect due to aseptic loosening of a cup (arthroplasty)	TCP granules (3 x 10 cc Cerasorb® M 5000-8000µm) & 10ccm Triosite® (60% HA, 40% TCP	Change of the acetabular component of a hip arthroplasty	+	
2	5	Finger enchondroma and pathological fx	TCP granules (1x 10 cc Cerasorb® M 3000-5000µm)	ORIF (plate/screws)	+	
4	6	Failed ankle pseudarthrosis	TCP granules (2 x 10 cc Cerasorb® M 1000-2000µm)	Arthrodesis of the ankle	+	
4	7	Failed ankle arthrodesis and pseudarthrosis	TCP granules (1 x 10 cc Cerasorb® M 5000-8000µm	Removal of the plate and arthrodesis with nail	+	
5	8	Significant bone loss due to fx of the proximal tibia and fibula	TCP granules (2 x 10 cc Cerasorb® M 3000-5000µm)	ORIF (plate/screws)	+	
3	9	Condition after Salter osteotomy and pseudarthrosis in DDH	1 OP Cerasorb® FOAM Flex	Modified osteotomy and osteosynthesis with a threaded nail	-	
5	10	Bone loss due to tibia & fibula fx	Cerament G, 1 OP Cerasorb® FOAM Flex, BMP-2	Osteosynthesis of the Tibia with Intramedullary Nail	+	
5	11	Bone loss due to tibia plateau fx	TCP granules (1 x10 cc Cerasorb® M 3000 - 5000µm)	ORIF (plate/screws)	+	
3	12	Thorax pseudarthrosis (rip/sternum junction)	1x Cerasorb® FOAM Flex	Resection, reconstruction	+	
1	13	Femoral fx by hip arthroplasty	TCP granules (1 x 10 cc Cerasorb® M 3000-5000µm)	Osteosynthesis with plate and screws	+	
4	14	Ankle arthrodesis and pseudarthrosis	TCP granules (1x 10 cc Cerasorb® M 5000-8000µm)	Arthrodesis (nail)	+	
4	15	Pseudarthrosis of the fibula	1x Cerasorb® FOAM Flex	ORIF (plate/screws)	+	
5	16	Bone defect due to fx of the acetabulum	1X Cerasorb® FOAM Flex	ORIF (plate/screws)	+	
5	17	Posttraumatic bone loss after tibia and fibula fx	TCP granules (1x 10 cc Cerasorb® M 5000-8000µm)	ORIF (plate/screws)	+	

1	18	Failed osteosynthesis with a bone defect proximal femur (γ-nail-cut-out)	TCP granules (1x 10 cc Cerasorb® M 5000-8000µm)	Hip arthroplasty	+
5	19	Fx of the distal humerus	TCP granules (1x 10 cc Cerasorb® M 1000 - 2000µm)	ORIF (plate/screws)	+
4	20	Posttraumatic osteonecrosis of the talus	TCP granules (2x 10 cc Cerasorb® M 5000-8000µm)	Arthrodesis with screws	+
5	21	Fx of the tibial plateau	TCP granules (1x 10 cc Cerasorb® M 3000 - 5000µm)	ORIF (plate/screws)	+
4	22	Pseudarthrosis of the fifth metatarsal	TCP granules (1x 10 cc Cerasorb® M 1000 - 2000µm)	ORIF (plate/screws)	+
5	23	Peritrochanteric femoral fx in a resurfacing hip	TCP granules (2x 10 cc Cerasorb® M 5000 - 8000µm)	TH revision, local treatment of the femoral bone defect	+
4	24	Osteoarthritis and instability of the ankle	Triosite®	Arthrodesis by nail	+
4	25	Pseudarthrosis of the ankle Joint	Triosite®	Fibula resection and arthrodesis with plate and screws	+
3	26	Necrosis of the head of femur	1x Cerasorb® FOAM Flex	Hip arthroplasty	+
3	27	Genu valgum	TCP granules (1x 10 cc Cerasorb® M 3000 - 5000µm)	Pelvic osteotomy and osteosynthesis with plate and screws	+
1	28	Loosening and dislocation of a cemented acetabular component of a hip arthroplasty	TCP granules (1x 10 cc Cerasorb® M 5000 - 8000µm)	Change of the acetabular component of a hip arthroplasty	+
4	29	Pain post-osteosynthesis with a γ-Nail	TCP granules (2x 10cc Cerasorb® M 5000 - 8000µm)	Explantation of a γ-nail and filling of the bone defect with TCP	+
3	30	Necrosis of the femoral head due to dysplastic fibrosis	TCP granules (1x 10cc Cerasorb® M 5000 - 8000µm) 1x allogenic cancellous bone chips	Hip arthroplasty and filling of a femoral cyst with TCP	+
4	31	Fx of the proximal humerus and osteosynthesis with plate and screws, Break of the screws and loosening of the plate	1x allogenic cancellous bone chips	Osteosynthesis with intramedullary humeral nail	+
5	32	Fx of the tibia plateau	Allogenic spongiosa	Osteosynthesis with plate and screws	+
5	33	Fx of the tibia plateau and of the proximal fibula	TCP granules (1x 10 cc Cerasorb® M 3000 - 5000µm)	ORIF (plate/screws)	+
3	34	Necrosis of the femoral head	TCP granules (2x 10 cc Cerasorb® M 5000 - 8000µm)	Hip arthroplasty	+
2	35	Enchondroma Dig. Pedis II	TCP granules (1 x 10 cc Cerasorb® M 5000 - 8000µm)	Curettage and filling up the defect with TCP	+
4	36	Fx of the clavicle and pseudarthrosis after an osteosynthesis with plate and screws	TCP granules (1x 10cc Cerasorb® 3000 - 5000µm)	ORIF (plate/screws)	+
2	37	Enchondroma Dig. V	TCP granules (1x 10cc Cerasorb® M 5000 - 8000µm)	Curettage and filling up the defect with TCP	+
5	38	Fx of the tibia plateau	TCP granules (1x 10 cc Cerasorb® M 5000 - 8000µm)	ORIF (plate/screws)	+
5	39	Fx of the tibia plateau	TCP granules (1x 10 cc Cerasorb® M 3000 - 5000µm)	ORIF (plate/screws)	+
5	40	Dislocation fx of the ankle joint	10 ml Cerament G	ORIF (intramedullary nail)	+
5	41	Fx of the tibia plateau	TCP granules (2x 10 cc Cerasorb® M 5000 - 8000µm)	ORIF (plate/screws)	+
5	42	Fx of the tibia plateu	TCP granules (2x 10cc Cerasorb® 3000 - 5000µm)	ORIF (plate/screws)	+

4	43	Sub-acute fx of the clavicle	1x Cerasorb® FOAM Flex	ORIF (plate/screws)	+
5	44	Fx of the tibia plateu	Allograft	ORIF (plate/screws)	+
5	45	Fx of the tibia plateu	TCP granules (2x 10cc Cerasorb® 3000 - 5000µm)	ORIF (plate/screws)	+
4	46	Patient after fx of the femoral neck and stabilizing with DHS	TCP granules (2x 10cc Cerasorb® 3000 - 5000µm) and 1x Cerasorb® FOAM Flex	Explantation of the DHS and curettage and filling up the defect with TCP	+
2	47	Pathological fx of D II by underlying enchondroma	TCP granules (1x 10cc Cerasorb® 3000 - 5000µm)	Curretage and filling up the defect with TCP, ORIF (plate/screws)	+
5	48	Fx of the tibia plateau	1x Cerasorb® FOAM Flex and allogenic bone	ORIF (plate/screws)	+
5	49	Pseudarthrosis of the proximal femur after fx and osteosynthesis with γ-Nail	1x Cerasorb® FOAM Flex	Resection of the pseudarthrosis and re-ORIF (plate/screws)	+
5	50	Arthritis of the ankle joint	1x Cerasorb® FOAM Flex	Arthrodesis with screws	+
